# HoloFood Data Portal: holo-omic datasets for analysing host–microbiota interactions in animal production

**DOI:** 10.1093/database/baae112

**Published:** 2025-01-11

**Authors:** Alexander B Rogers, Varsha Kale, Germana Baldi, Antton Alberdi, M Thomas P Gilbert, Dipayan Gupta, Morten T Limborg, Sen Li, Thomas Payne, Bent Petersen, Jacob A Rasmussen, Lorna Richardson, Robert D Finn

**Affiliations:** European Bioinformatics Institute (EMBL-EBI), European Molecular Biology Laboratory, Wellcome Genome Campus, Hinxton, CB10 1SD, UK; European Bioinformatics Institute (EMBL-EBI), European Molecular Biology Laboratory, Wellcome Genome Campus, Hinxton, CB10 1SD, UK; European Bioinformatics Institute (EMBL-EBI), European Molecular Biology Laboratory, Wellcome Genome Campus, Hinxton, CB10 1SD, UK; Center for Evolutionary Hologenomics, Globe Institute, University of Copenhagen, Øster Farimagsgade 5, Copenhagen 1353, Denmark; Center for Evolutionary Hologenomics, Globe Institute, University of Copenhagen, Øster Farimagsgade 5, Copenhagen 1353, Denmark; University Museum, NTNU, Vitenskapsmuseet, Trondheim 7491, Norway; European Bioinformatics Institute (EMBL-EBI), European Molecular Biology Laboratory, Wellcome Genome Campus, Hinxton, CB10 1SD, UK; Center for Evolutionary Hologenomics, Globe Institute, University of Copenhagen, Øster Farimagsgade 5, Copenhagen 1353, Denmark; Center for Evolutionary Hologenomics, Globe Institute, University of Copenhagen, Øster Farimagsgade 5, Copenhagen 1353, Denmark; European Bioinformatics Institute (EMBL-EBI), European Molecular Biology Laboratory, Wellcome Genome Campus, Hinxton, CB10 1SD, UK; Center for Evolutionary Hologenomics, Globe Institute, University of Copenhagen, Øster Farimagsgade 5, Copenhagen 1353, Denmark; Centre of Excellence for Omics-Driven Computational Biodiscovery (COMBio), Faculty of Applied Sciences, AIMST University, Batu 3 1/2, Jalan, Bukit Air Nasi, Bedong, Kedah 08100, Malaysia; Center for Evolutionary Hologenomics, Globe Institute, University of Copenhagen, Øster Farimagsgade 5, Copenhagen 1353, Denmark; European Bioinformatics Institute (EMBL-EBI), European Molecular Biology Laboratory, Wellcome Genome Campus, Hinxton, CB10 1SD, UK; European Bioinformatics Institute (EMBL-EBI), European Molecular Biology Laboratory, Wellcome Genome Campus, Hinxton, CB10 1SD, UK

## Abstract

The HoloFood project used a hologenomic approach to understand the impact of host–microbiota interactions on salmon and chicken production by analysing multiomic data, phenotypic characteristics, and associated metadata in response to novel feeds. The project’s raw data, derived analyses, and metadata are deposited in public, open archives (BioSamples, European Nucleotide Archive, MetaboLights, and MGnify), so making use of these diverse data types may require access to multiple resources. This is especially complex where analysis pipelines produce derived outputs such as functional profiles or genome catalogues. The HoloFood Data Portal is a web resource that simplifies access to the project datasets. For example, users can conveniently access multiomic datasets derived from the same individual or retrieve host phenotypic data with a linked gut microbiome sample. Project-specific metagenome-assembled genome and viral catalogues are also provided, linking to broader datasets in MGnify. The portal stores only data necessary to provide these relationships, with possible linking to the underlying repositories. The portal showcases a model approach for how future multiomics datasets can be made available.

**Database URL:**  https://www.holofooddata.org

## Introduction

Optimizing the production of food is a major challenge for the world’s growing population, as highlighted by the emphasis on agricultural and aquacultural developments in the United Nation’s Sustainable Development Goals [1] and the European Commission’s European Union (EU) Missions [2]. Animal production for food is a crucial area for development since the requirements of farming both animals and their feedstock have compound effects on land and marine use. In this context, microbiome-based solutions are the subject of intense research as a mechanism to optimize the conversion of feeds, understand the impact of alternative feeds, and improve the health and yield of farmed animals [3]. Despite this attention, our understanding of how microbial communities aid digestive processes and interact with their animal hosts is still very limited, and animals are rarely conceptualized as host–microbiota systems (or holobionts) by producers.

The international project HoloFood (https://www.holofood.eu) was funded through the EU’s Horizon 2020 Research and Innovation Actions and implemented a holo-omic approach [4], involving the generation and analysis of multiple omic layers for both the host animal and the associated microbiome. This unique effort aimed at better understanding how animal–microbiota interactions affect animal production under different feed conditions. To address the global twin challenges of land and marine usage for food, the HoloFood project targeted two radically different animal systems: poultry [5] and salmon [4]. Chicken (*Gallus gallus*) poultry is produced from an avian terrestrial system in which animals typically live < 12 weeks, whereas salmon (*Salmo salar*) is produced in an aquaculture system where animals are grown for around 3 years before harvest. Diverse feed interventions were also used for the two systems, with the addition of prebiotics and probiotics being the focus of investigation for poultry [5], while the salmon studies investigated the use of seaweed and blue mussel as novel ingredients in the fish feed [4].

The HoloFood consortium conducted seven different trials towards these goals (three for chicken and four for salmon). Multiple samples were collected from each animal, and these were analysed to characterize the biological properties of each holobiont (the animal and its microbiota). A typical animal provided four samples, but 157 animals provided 10 or more samples each. A multiomic experimental design was adopted whereby each sample contributed to determining one or more omic layers of each holobiont system: the host genome, host transcriptome, microbial metagenome, microbial metatranscriptome, or the metabolome. In addition, a detailed metadata collection process was paired with quantitative production indicators to build a hierarchical picture of each system through measurements at multiple levels: the individual animal, the pen or tank, and the trial.

This experimental design—necessary to understand the complexity of holobionts—resulted in a large and complex dataset. The HoloFood consortium deposited each dataset into appropriate dedicated public repositories following international data standards. These were the European Nucleotide Archive (ENA) for nucleic acid data [6], MetaboLights for metabolomic data [7], and BioSamples for metadata and production metrics [8]. Assemblies and annotations based on the analysis of the microbiome-associated raw sequences were stored in MGnify [9], while structured data for molecular assay results like fatty acids and inflammatory markers were added to the relevant BioSample entries.

Nevertheless, the complex HoloFood data model is not easily represented in these aforementioned repositories. For example, ENA contains metadata related to nucleotide reads and defines relationships between data objects that conform to the International Nucleotide Sequence Database Collaboration (INSDC) data model: namely, overarching studies, their samples, experiments, reads, and downstream derived datasets like genome assemblies. Likewise, MetaboLights follows an appropriate data model for metabolomics studies, represented by the Investigation, Study, and Assay framework. Linking related datasets between these different contexts, and different data models, is a challenge that the BioSamples repository helps to facilitate. BioSamples provides a generic system for describing biological samples, along with a relationship model to link one or more samples derived from or equivalent to one another. ENA, MGnify, and MetaboLights all support the BioSamples accessioning scheme, thus facilitating the linkage of these three resources. This relationship model and broad support for their accessioning scheme make BioSamples accessions an ideal unique reference for the animals and samples produced by HoloFood. Despite being the canonical identifier of the samples and relationships, BioSamples is a general repository and so cannot represent the specific details of each intersample relationship nor display the myriad of datasets in other repositories that relate to a given BioSample. This makes it hard for the user to discover the multiomic data that may be associated with a sample since it requires several searches in multiple repositories. For example, co-analysing the microbial taxonomic diversity of probiotic-fed chicken gut metagenomes with the corresponding host genome reads for the same animal would require searching three repositories (BioSamples, ENA, and MGnify).

Here, we present the HoloFood Data Portal, a web resource we developed to overcome this complexity and support the findability of inter-related data archived in multiple repositories. It presents an organized view of the primary multiomics data of the HoloFood project and federates queries to the canonical data repositories for each omic type. This showcases a model approach to making complex project datasets more findable, accessible, interoperable, and reusable [10] (FAIR), by providing a layer of interconnectivity based on the underlying experimental design.

## Methods

### BioSamples-based schema design

**Figure 1.**
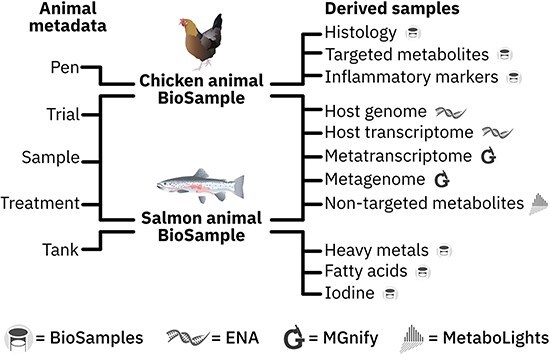
The schema used for HoloFood animals and samples registered in BioSamples [8]. Individual animals are accessed as BioSamples, with metadata sections as shown on the left. Most of these metadata sections are shared between the two systems. Extraction-level (derived) samples are registered separately in BioSamples, and BioSamples relationships are used to connect each derived sample to its parent animal-level sample. Metadata are also available on the derived samples, which include the experimental results for assays like fatty acid measurements in salmon and inflammatory markers in chicken. Analysis results for the derived samples are stored in the most appropriate repository, as indicated by the service icons alongside each type.

One of the most fundamental choices made in depositing the HoloFood dataset was the choice of the BioSamples schema. The adopted schema (see Figure 1) is based on a two-level hierarchy of animal–sample (or host extraction) for accessing and linking the BioSamples, with structured metadata attached to each. Trial, enclosure (pen or tank), and host-level metadata like trial conditions and performance metrics are attached as structured metadata to the animal-level BioSamples objects. Sampling information, e.g. extraction location within the gut, is attached to the derived BioSamples objects. At the extraction level, each BioSample represents a single biological sample collected for a specific purpose in the overall experimental design. These purposes are presented as ‘sample types’ in the data portal, with each type having an internally consistent metadata schema.

Where experimental results were obtained that do not logically fit in any pre-existing repository (e.g. fatty acid, heavy metals, iodine, and inflammatory marker measurements), these are also attached as structured metadata, in an appropriately titled group, to the relevant BioSample. BioSamples also supports the addition of external links to samples, and MetaboLights MTBLS study references have been added to metabolomic samples in BioSamples.

### Data loading into the HoloFood Data Portal

The data portal is designed to store the minimum amount of information possible, with a preference for linking to data stored in the most appropriate public repositories to ensure availability, while minimizing data duplication.

The data loading process begins by fetching a list of animal- and extraction-level BioSamples, using an Application Programming Interface (API) query with the filter attr: project=HoloFood and limiting to samples submitted by the accounts associated with the HoloFood project. The BioSamples structured metadata contains data necessary for many queries and for linking samples to external repositories. For this reason, the sample metadata are duplicated (effectively cached) in the data portal database. From these metadata, the data portal can check external services—MetaboLights and MGnify—for the existence of related datasets on metabolomic and metagenomic sample types, using their public APIs (https://www.ebi.ac.uk/metabolights/ws and https://www.ebi.ac.uk/metagenomics/api, respectively). The HoloFood Data Portal thus contains a relational model of animals, samples, metadata, and external identifiers for the HoloFood sample set.

The HoloFood Data Portal is also the canonical database hosting some of the project’s derived datasets: genome catalogues, viral fragment catalogues, and analysis summaries. The catalogues are loaded from TSV files, and the summaries are authored by project partners in an administrative interface of the data portal.

### Relationships to cross-project catalogues

The genome catalogues in the HoloFood Data Portal are subsets of wider cross-project catalogues produced by MGnify using their biome-specific metagenome-assembled genome (MAG) catalogue generation workflow [11]. When the MGnify catalogues are generated, genomes are quality controlled and clustered into species-level groups using an average-nucleotide identity threshold. The best quality genome from each cluster is selected as the species representative, and only these representative genomes are shown on the MGnify Genomes website [11].

**Figure 2.**
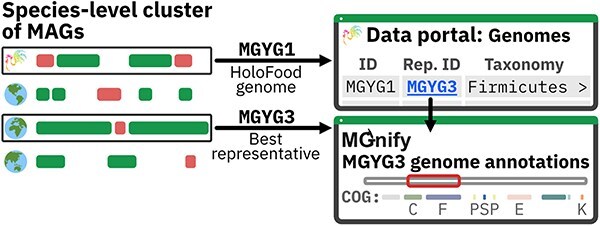
Genome catalogues were created for each system. HoloFood-derived metagenome-assembled genomes were combined with other publicly available chicken and fish data to produce genome catalogues for the MGnify Genomes resource [11]. In this example, the HoloFood genome clusters with three other genomes at the species level. This genome is best represented by a non-HoloFood representative. The data portal lists the HoloFood-derived genomes, with links to the best cluster representative for each species, for which annotations can be browsed on MGnify.

To avoid duplicating the data and interfaces of MGnify, the data portal simply lists the MGnify Genome accessions of all HoloFood-derived genomes and links to their cluster representative on MGnify (Figure 2). However, given the importance of carbohydrate-active enzymes to interpreting HoloFood results, Carbohydrate-Active enZyme (CAZyme) annotations are available in the data portal directly for each MAG (based on its cluster representative). This cluster representative may be the HoloFood-derived genome itself or a higher-quality representative from another dataset. In all cases, the assumption is that many users will find the genome annotations available for the cluster representative on MGnify to be useful for a given HoloFood genome. When this is not the case, e.g. when there is a need for strain-level information that makes the representative unsuitable, users can obtain annotations for the HoloFood genome itself from the MGnify FTP server locations linked to by the catalogue webpages.

To build the sample-containment list for each MAG, the sequence of its cluster representative MAG was queried against the entire project’s metagenomic reads. This query was conducted using Mastiff, based on sourmash [23], using a kmer length of 21.

### Federated API design (canonical URLs)

For users accessing the data portal programmatically via the API, the design choice to link out as much as possible to appropriate repositories is implemented as a series of canonical URLs and omics-type specific URLs. The canonical URLs point to the most appropriate programmatically accessible source for each data object. These are the ENA Portal API for genomic, transcriptomic, metagenomic, and metatranscriptomic read samples, the BioSamples API for metabolomic and phenotypic samples as well as animals, and the data portal itself for analysis summaries. Additional URLs are returned for metagenomics analyses (from the MGnify API) and for metabolomic analyses (from the MetaboLights API).

The data portal API does not attempt to proxy requests to these repository APIs. Given that they use a myriad of technologies and formats and are all routinely updated and likely to evolve significantly in their format over the lifetime of the data portal, we chose to keep the data portal easily maintainable by only providing the API URLs for objects, which are relatively easily updated. Many of the repository APIs use (different) standards and are self-describing, but users should consult each API’s documentation for details. The data portal documentation includes basic examples of how to programmatically access the data portal and these external APIs together.

### Portal technologies

Our goal was to create an easily maintainable codebase for the data portal that can serve the research community for several years after the project is completed. In line with this, we adopted widely used, mature technologies. The data portal is a Django (Python) application, backed by a SQLite database. Django provides the Object Relational Manager (ORM) for the database, an administrative interface, a framework for management commands used to import data, and page templating and rendering used for the website. Django Ninja was chosen to provide the API and TSV Export functionality, due to its low code footprint and ability to share the ORM with the website. Pydantic has been used for schema-checked and type-checked configuration file handling, to help future developers unfamiliar with the codebase to make small changes without introducing bugs. These choices have made the codebase relatively portable between web hosting environments, e.g. we have deployed it to both AWS Elastic Beanstalk and Kubernetes clusters.

## Results

**Figure 3.**
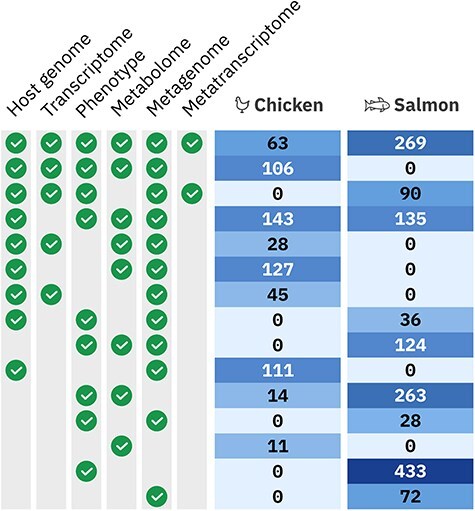
Overview of the multiomic samples available in the HoloFood Data Portal for each system. Different multiomics sample sets were extracted from animals in multiple trials and time points. Each row shows how many individual animals are available covering that multi-omic combination. Combinations are shown where at least five individuals are available (which excludes eight chickens and two salmon). ‘Phenotype’ encompasses several experiment types: inflammatory markers, histology, fatty acids, iodine, and heavy metals; half of the 433 salmon with only phenotypic data have more than one phenotypic sample type.

In total, 9889 samples were extracted from 2108 animals across seven trials, collectively described by 841 928 metadata entries. The hologenomic data alone consist of 112 billion base pairs of genomic and transcriptomic data, and the metabolomic mass spectrometry data total 279 GB. Moreover, the holo-omic design results in a richly connected multiomics dataset, with 1809 of animals having multiple omics layers (Figure 3). The HoloFood Data Portal organizes these samples and their inter-relationships into a relational database where it is possible to query for multiomic data from the same host or hosts in the same experimental group. This structure is the base level of data organization required to make use of an experimental holo-omic dataset (Figure 1).

The data portal also stores a cache of the rich metadata associated with these samples. With sample-derived datasets being hosted in the most appropriate repository for their type, the data portal stores the accessions, identifiers, and URLs to access processed information about genomic and transcriptomic reads (in ENA), metabolites (in MetaboLights), and microbial communities (in MGnify). HoloFood datasets are also integrated into broader cross-project datasets, like MGnify’s biome-specific microbial genome catalogues for chicken and nonmodel fish gut [11], where these can be contextualized, allowing diversity and novelty to be explored. The MAGs derived from HoloFood samples are listed in the data portal along with links to their closest species representative genome in the wider MGnify biome-specific catalogues.

Large projects like HoloFood generate information beyond the standard structured datasets that can be deposited in the established archives. Our approach to capturing and preserving additional notes, observations, and discoveries is to document them in an ‘annotation layer’ on the HoloFood Data Portal, organized into ‘analysis summaries’. Each summary is a formatted text document tagged to one or more related samples or catalogues. This layer allows consortium partners and collaborators to share relevant information with the community in a form that sits between the metadata submitted to repositories and the research findings communicated in publications. The analysis summaries are stored as Markdown documents in the data portal database. These are rendered as HTML for users of the data portal website or can be downloaded for conversion into other forms.

**Figure 4.**
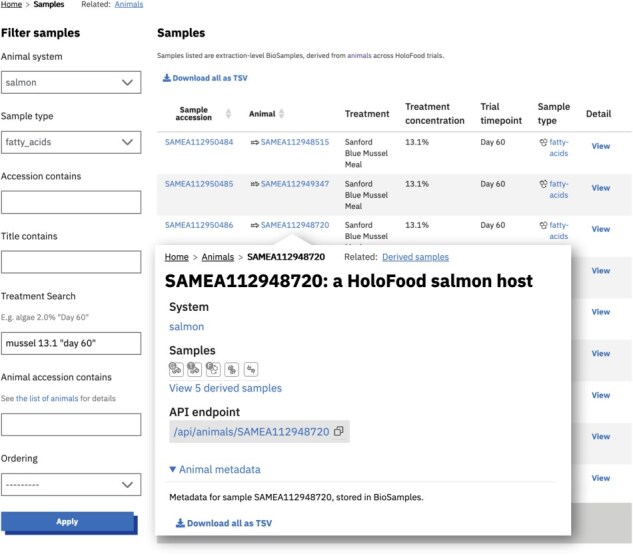
The primary dataset available in the data portal is a list of samples (registered in BioSamples). The sample list (in the background of this composite screenshot) can be partitioned along different axes: in this case by system, omic type, and treatment. It can also be partitioned by host, as shown by the inset which shows the content of a salmon detail page linking to five derived omics samples. Animals (which are also represented by a BioSamples identifier) can be similarly listed and the hierarchical relationship between samples and animals can be used to navigate and filter the data. The same filters and lists can be used programmatically via the API.

**Figure 5.**
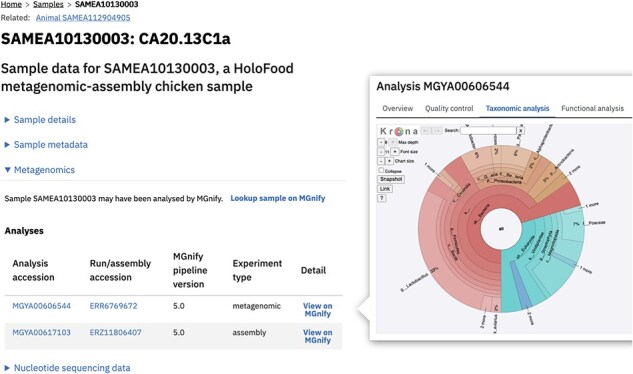
Screenshot showing a sample detail page for a metagenomic sample (left). The analysis of metagenomic samples is performed by and stored in MGnify [9]. The data portal shows a summary of this MGnify data and links to MGnify where users can browse the analysis dataset (right).

These datasets—the samples and their metadata, the identifiers and metadata of derived datasets, the contributions to cross-project catalogues, and the analysis summaries—are accessible (i) via the data portal website, (ii) via the API, and (iii) as database snapshot files. The data portal can be used by researchers interested in HoloFood’s data legacy to query for and access multiomic slices of the data. Filters are available to find samples by their type and host/trial condition (Figure 4). Each sample’s detail page links to the data in its canonical repository, and the website also presents summaries of the analyses available in those repositories. For data in ENA, the related runs and experiments are tabulated; for data in MetaboLights, the raw and derived files are listed; and for data in MGnify, the sample’s metagenomic analyses are listed (Figure 5).

MGnify provides biome-specific MAG catalogues, two of which are supersets of the HoloFood data, namely, the chicken gut and nonmodel fish gut MAG catalogues. The MGnify Genomes website presents species-representative genomes and their pan-genomes, annotated with taxonomies, predicted coding sequences, noncoding RNAs, biosynthetic gene clusters, and viral regions [11]. These sequences have functional annotations including InterPro identifiers [12], Clusters of Orthologous Gene categories [13], and Kyoto Encyclopedia of Genes and Genomes (KEGG) orthologies [14]. MAGs constructed from HoloFood data are listed in the HoloFood Data Portal and link to the relevant annotated species representatives in MGnify (Figure 2). Phylogenetic trees were produced for the HoloFood subsets of each catalogue, and these are available in the data portal as analysis summaries linked to the catalogues.

Given the HoloFood project’s focus on novel feeds, the CAZyme annotations available for the MGnify MAG catalogues are particularly relevant. The data portal therefore includes a summary of the CAZy [15] annotations for each MAG, based on annotations of its cluster representative. These summaries are counts of the annotations in each of the six CAZy categories. The data portal also includes a list of samples in which each MAG is present and the level of containment for each case. This allows data portal users to find metagenomic samples that contain a MAG’s kmers above a certain minimum threshold. Consequently, users can find sample metadata such as trial conditions associated with the presence of MAG species and, in turn, the prevalence of CAZyme annotations, as well as any other related sequence data for the same animal and sample.

During preparation of the metagenomic datasets, contigs were scanned for viral signatures [16]. At present, MGnify supports the browsing of viral annotations on metagenomic assemblies and MAGs. However, these viral sequences are not currently clustered into representative viral catalogues. The data portal therefore includes lists of clustered viral sequences discovered in the HoloFood metagenomic samples. These viral predictions can be viewed in the data portal directly, which loads the associated contig from the MGnify API and shows the predicted viral region using the Integrative Genomics Viewer browser [17]. The URLs and General Feature Format content required to reproduce these visualisations are also available via the data portal’s API.

### Use case: multiomic analysis of carbohydrate utilization in poultry

A researcher is interested in understanding the difference in carbohydrate utilization of different breeds of poultry. Using the HoloFood Data Portal, the researcher can obtain a multiomic dataset for this analysis. First, they fetch three sample lists, all filtered by ‘system: chicken’ and by ‘sample type: metagenomic_assembly’, ‘sample type: metatranscriptomic’, and ‘sample type: metabolomic_targeted’, respectively. They find the intersection of these three sample lists by matching the samples’ animal-level BioSamples accession, leaving 66 animals with all three sample types of interest. They use the portal’s animal detail views to find the ‘Breed’ metadata, segmenting the dataset into 24 Cobb and 42 Ross animals.

For the metagenomic and metatranscriptomic samples, the portal returns a ‘metagenomics URL’, to lookup the sample in MGnify [9]. The MGnify API’s hyperlinked relationships can then be followed to find the sample’s functional analysis results, where KEGG Ortholog counts [14] and the completeness of relevant KEGG modules related to carbohydrate metabolism can be retrieved. The results of targeted metabolomics assays, such as measurements of lactate concentrations and ratios, are stored in the BioSamples structured data and are therefore available from the data portal on each metabolomic samples’ detail view.

This combined multiomic dataset can be reshaped into a feature table of sample (animal), group types (breed and trial), and measurements (module completeness, relative abundance in metatranscriptomics, and metabolomic concentrations). This feature table can then be analysed using multiomics factoring tools like Multi-Omics Factor Analysis [18] to determine whether and what statistically significant differences exist between the groups.

## Discussion

Good data stewardship requires that datasets like this be deposited appropriately into widely recognized repositories and that the complex experimental design is not lost or only found buried in publications in nonmachine readable formats. Producing and analysing multiomic data is costly and time-consuming, yet the scientific and sustainability benefits of further use and reanalysis of a dataset like HoloFood’s are many. The tools to integrate and model such complex project designs are only now becoming available, and to this end, the HoloFood Data Portal ensures that the design is captured and accessible to both humans and machines for downstream analysis.

HoloFood’s data are also foundational for emerging projects, including the EU Horizon-funded projects FindingPheno (DOI: 10.3030/952 914, https://findingpheno.eu) and BlueRemediomics (DOI: 10.3030/101 082 304, https://blueremediomics.eu). Therefore, there are immediate benefits to ensuring that the project’s data are not just deposited, but the relationships between samples and data types are also conveniently accessible and well described. All potential users of the dataset will require accurately captured contextual information in the form of sample metadata, an understanding of the study design, and clearly defined relationships between the multiomic data.

The HoloFood Data Portal can be considered an extension of the existing project-wide summary provided by NCBI BioProject. HoloFood has registered an INSDC Umbrella Project (PRJEB43192) which can be browsed on the BioProject website. However, this is an INSDC resource and therefore limited to showcasing all the nucleotide data generated by the project. Notably, this omits non-nucleotide data (like metabolomic information stored in MetaboLights, histological and performance metric data listed in BioSamples Structured Metadata, and metagenomics analyses stored in MGnify). Moreover, BioProject cannot represent the sample hierarchies of holo-omic data where those hierarchies are dependent on the experimental design.

The feature set of the HoloFood Data Portal has many parallels with those of FAIRDOM-SEEK [19, 20] and Dataverse [21], which are platforms for creating research data management hubs (including a database, website, and API). For many multiomic projects, these platforms could be deployed to provide the necessary data model and interfaces to meet a project’s internal needs of a data management system and provide public access to the project’s data following FAIR principles. For HoloFood, a bespoke data portal approach was chosen to visualize the multiomic and holo-omic relationships as clearly as possible and to minimize duplication between the canonical data repositories and the data management system itself. In future, we expect more interconnectivity between platforms such as FAIRDOM-SEEK and the different omics repositories, and recent work has shown the potential for automatic deposition of data stored in FAIRDOM-SEEK DataHubs into ENA [22].

Large-scale multiomic datasets like the one described here are set to become ever more common as scientists try to understand the complex interplay between host and microbiome, in the context of phenotypes and the environment. With diminishing sequencing costs fueling the growth of such projects, this will pose a challenge for researchers looking to study these datasets and for repository developers needing to generalize the data model across a wide range of disciplines and experimental designs. The HoloFood Data Portal showcases a model for how such projects can follow best practice in terms of data deposition, while adding a project-aware query layer that reflects the project design and improves data FAIRness, without duplicating the content and remit of specific omics repositories.

## Author contribution

Alexander B. Rogers (Software, Methodology, Resources, Writing—original draft), Varsha Kale (Data curation, Methodology), Germana Baldi (Data curation, Visualization), Dipayan Gupta, Sen Li, Thomas Payne, Jacob A. Rasmussen, Lorna Richardson (Data curation), and Antton Alberdi, M. Thomas P. Gilbert, Morten T. Limborg, Robert D. Finn (Conceptualization, Supervision, Writing—review and editing, Funding acquisition).

## Data availability

The HoloFood Data Portal (website and API) is available at https://www.holofooddata.org. Documentation is available at https://docs.holofooddata.org. Snapshots of the data portal’s database are available as SQLite files at https://ftp.ebi.ac.uk/pub/databases/metagenomics/holofood_data. Animal and extraction-level samples and metadata have been deposited in the BioSamples repository at the European Molecular Biology Laboratory’s European Bioinformatics Institute (EMBL-EBI) and can be queried using the project=HoloFood attribute in https://www.ebi.ac.uk/biosamples. Nucleic acid data have been deposited in the ENA at EMBL-EBI under umbrella project accession number PRJEB43192 (https://www.ebi.ac.uk/ena/browser/view/PRJEB43192). Metagenomic data are available from MGnify at EMBL-EBI, under the Super Study ‘holofood’ (https://www.ebi.ac.uk/metagenomics/super-studies/holofood). Metabolomic data are available from MetaboLights at EMBL-EBI (https://www.ebi.ac.uk/metabolights) in studies MTBLS4381, MTBLS4382, MTBLS4384, MTBLS6733 (Salmon), and MTBLS6988 (Chicken). The data portal’s codebase is permissively licensed (Apache 2.0) and is available on Zenodo (https://zenodo.org/records/12 698 157, DOI: 10.5281/zenodo.7684071) and GitHub (https://www.github.com/ebi-metagenomics/holofood-database). Workflows used by the HoloFood consortium to produce the deposited data are available at https://workflowhub.eu/programmes/28.
